# Characteristics of digital twins to visualize, monitor and predict outcomes on participation in meaningful activities

**DOI:** 10.3389/fpsyt.2025.1694966

**Published:** 2026-01-28

**Authors:** Vera C Kaelin, Kaan Kilic, Anna Stigsdotter Neely, Hanna M. Gavelin, Patrik Wennberg, Helena Lindgren

**Affiliations:** 1 Department of Computing Science, Umeå University, Umeå, Sweden; 2 Department of Health, Education and Technology, Luleå University of Technology, Luleå, Sweden; 3 Department of Psychology, Umeå University, Umeå University, Umeå, Sweden; 4 Department of Public Health and Clinical Medicine, Umeå University, Umeå, Sweden

**Keywords:** occupational therapy, recovery experiences, stress, exhaustion, technology, engagement, activity performance, mental health

## Abstract

**Introduction:**

This study aimed to explore the perspectives of occupational therapists and individuals with lived experience of mental health challenges regarding which aspects of participation in meaningful activities should be represented in digital twins, and how these aspects should be visualized.

**Methods:**

We conducted a qualitative descriptive study involving 14 semi-structured interviews with occupational therapists and individuals with lived experience of mental health challenges. Interviews were transcribed verbatim and analyzed using inductive content analysis.

**Results:**

Participants identified four main aspects to visualize in digital twins focusing on participation in meaningful activities: 1) the degree of being present and in-the-moment, 2) related or resulting experiences of being present and in-the-moment (including relaxation and calmness; fulfillment, purpose, joy, and playfulness; feeling vitalized; connection with oneself, others, and the world), 3) the energy needed and the energy gained from participation, and 4) the influence of the context. In addition, results revealed two main approaches for how these aspects should be visualized: 1) symbols and charts to visualize the now-situation, and 2) graphs for visualization over time.

**Discussion and conclusion:**

Results identified four aspects of a participation in meaningful activities to be represented in digital twins. Those align with, and extend, existing literature on participation and recovery experiences. For the visualization of those aspects in digital twins, participants emphasized the importance of integrating both immediate feedback and longitudinal tracking of participation experiences.

## 1 Introduction

Participation in meaningful activities is a key ingredient and outcome of occupational therapy services (1). It has been shown to positively impact individuals’ satisfaction (2), happiness (3), meaning in life (2), and recovery from mental health challenges (4). However, participation is frequently and significantly lower among people with lived experience of disability versus those without (5, 6), including individuals with mental health challenges (7).

Participation has been defined in the family of participation-related constructs (fPRC) framework as comprising two dimensions: attendance (being there) and involvement (the subjective experience of being there or doing an activity) (8). Meaningful activities have been described in various ways including psychological rewarding (9) or positive activities (10). Although definitions vary across these terms, they share a common emphasis on activities that provide personal meaning, alleviate negative stress, and support health and well-being. In this study we use the terms ‘meaningful activities’ to refer to those that carry individual significance and simultaneously generate positive affect and energy. What constitutes as a meaningful activity, however, varies broadly among individuals. For example, household tasks may introduce stress for some but are restorative and energy-giving for others.

In recent years, the concept of digital twin has emerged as a powerful tool for mirroring and understanding complex systems. A digital twin is a digital representation of a physical object, space, process, or living organism such as humans that enables visualization, monitoring, and prediction of real-world behavior (11). This is done by continuously gathering data (e.g., sensor data) from the real-world entity, which are being fed into the digital twin (11). Initially developed by NASA in the 1970s to mirror and monitor inaccessible physical spaces such as spacecraft (11), digital twins were later adopted in manufacturing for product life-cycle management (11) and have since gained increased interest across various domains, including healthcare (11).

In healthcare, digital twins are gaining interest for their potential to enable personalized care and predictive insights (11–13). They have been used to model physiological systems, predict treatment outcomes, and support health-related decision-making, often presented to the patients through visualization of the data. Recent developments emphasize the use of digital twins not only to replicate physical parameters (e.g., heart rate, blood pressure) but also to integrate behavioral and lifestyle data to support proactive health management (13). Such developments highlight the potential of digital twins to provide holistic representation of human health and well-being.

Despite this potential, the application of digital twins in mental health and occupational therapy remains underexplored. Existing healthcare digital twins largely focus on biological data and physical parameters (11–13), with limited attention to data related to participation in meaningful activities (14–16). This gap is significant given the importance of participation in meaningful activities for individuals’ health and well-being (17, 18). One possible reason for this oversight is a limited understanding of which aspects of participation should be visualized and how they should be represented to support individuals in reflecting on and managing their participation in meaningful activities. Given the complexity of the construct of participation in meaningful activities and its multifaceted relationship with health and well-being, relying solely on theoretical frameworks may not lead to the development of a tool that adequately reflects end users’ needs (19, 20). Moreover, digital twins often aim to visually replicate an object or process as accurately and realistically as possible, yet the experience of activity participation also encompasses subjective and partly invisible dimensions (e.g., individuals experience of involvement in meaningful activities (8)).

To address these knowledge gaps, the present study aimed to explore the perspectives of occupational therapists and individuals with lived experience of mental health challenges on which aspects of activity participation in meaningful activities should be represented in digital twins, and how these aspects should be visualized. This work contributes to bridging the fields of digital health technology and occupational therapy by informing the design of participation-focused digital twins that reflect both observable and experiential aspects of meaningful activity participation.

## 2 Materials and methods

### 2.1 Study design

We applied a qualitative descriptive study design (21). The research project was approved by the Swedish Ethical Review Authority (Dnr. 2019-02794).

### 2.2 Participants

Participants were occupational therapists and individuals with experience of mental health challenges. Occupational therapists have extensive knowledge on participation, meaningful activities, and their complex interactions with health and well-being (1). Individuals with experience of mental health challenges are considered as potential end users of a participation-focused digital twin. Participants were recruited between August and December 2024 via email, using purposive and snowball sampling within our professional networks. Individuals were included if they 1) had an official degree in occupational therapy or identified as individuals with current or past mental health challenges, 2) could read and speak English, and 3) had internet access.

### 2.3 Data collection

**Figure 1:**
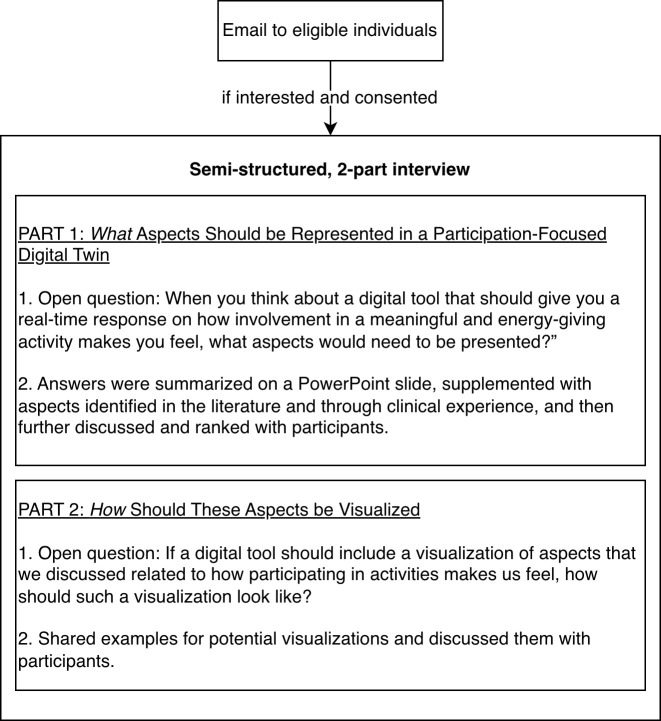
The steps of data collection.

The research staff sent an email to eligible individuals with information about the study (see **Figure 1**). If individuals were interested in participating, research staff invited them to an online semi-structured interview via Microsoft Teams. Interviews lasted 36–80 minutes and were conducted by the first author (VK) who has prior experience with qualitative research. The interview consisted of two parts. Part 1 focused on *what* aspects participants would like to have represented and visualized in a digital twin on participation in meaningful activities (i.e., those that carry individual significance and simultaneously generate positive affect and energy), and part 2 focused on *how* participants envision these aspects to be visualized in such a digital twin. For part 1, participants were asked to share insights on what aspects they would like to have visualized related to their participation in meaningful activities. For this, they were first asked an open question (“When you think about a digital tool that should give you a real-time response on how involvement in a meaningful and energy-giving activity makes you feel, what aspects would need to be presented?”). Answers of participants were summarized on a PowerPoint slide and then supplemented with aspects, identified by literature and clinical experience. This PowerPoint slide was then shared with participants to reflect on them. Participants were asked whether they would delete or add anything and how they would rank the identified aspects in terms of importance.

For part 2, participants were asked how they envision these aspects to be visualized. Participants were first asked the open question (“If a digital tool should include a visualization of aspects that we discussed related to how participating in activities makes us feel, how should such a visualization look like?”). Then, we shared examples of current visualizations in digital twins. More specifically, we shared an existing prototype using spiderweb visualization and examples (e.g., graphs, visualizations of tangible objects including a sun, clouds and rain as well as a tree with growing branches) from the Flutter-based fl_chart library^1^. The interview guide was pilot-tested before being finalized and used with interview participants. Interviews were audio-recorded and transcribed verbatim. Participant recruitment continued until new data did not yield significant new information related to the research question, signifying the attainment of data saturation.

### 2.4 Data analysis

We conducted a qualitative content analysis following the inductive approach described by Elo and Kyngäs (22) and Elo et al. (23). The analysis was performed by a single researcher and proceeded through three main phases: preparation, organizing, and reporting. In the preparation phase, the researcher read the transcripts multiple times to gain a comprehensive understanding of the data. The unit of analysis was set to be on sentence level. During the organizing phase Taguette software (24) was used. The analyzing researcher applied open coding with notes to capture key concepts while focusing on the manifest content in the transcript and ignoring the latent content (e.g., including data on moments of silence, laughter). These codes were then grouped into categories based on similarities and differences and interpretation of the data, allowing for the development of higher-order categories that reflected the core content of the data. Categories were checked by research team members for potential overlaps between them. In the reporting phase, the main categories and subcategories were described and supported by illustrative quotations from participants, ensuring transparency and rigor in the analytical process. Each category was named using content-characteristic words. This structured approach enabled a systematic exploration of participants’ perspectives on the visualization of meaningful activity participation in digital twins.

## 3 Results

### 3.1 Sample characteristics and types of meaningful activities

A total of 7 individuals with lived experience of mental health challenges and 7 occupational therapists participated in this qualitative research study. The age ranged from 29 to 44 years (mean = 34 years) among individuals with lived experience of mental health challenges and 26 to 45 years (mean= 36 years) among occupational therapists. Across individuals with lived experience and occupational therapists, ten out of 14 (71%; n=5 out of 7 for each participant group) identified as female and four as male. A total of eight out of 14 (57%; n=4 out of 7 for each participant group) had a master’s degree and six had a doctorate degree. They mentioned a broad range of activities to describe what kind of activities were meaningful and energy-giving to them. Examples included socializing, walking, dancing, meditating, exercising, and reading (see **Table 1**).

**Table 1:** Sample demographics and meaningful activities.

Participant		Technology usage	Meaningful activities
U1		Productivity timer, games to relax, sleep and activity tracker	Go outdoors with children, exercise, having a shower and coffee
U2		Meditation and sleep apps	Socializing with my husband, eating out with close friends or husband, listening to music or religious podcasts
U3		Activity tracker and health app	Going into the nature, creative activities, dancing, music, sketching, discussing ideas, activities with my pets
U4		None	Socializing activities in smaller groups and face-to-face, writing in journal
U5		Healthy minds	Meditation, reading, exercising, lifting heavy weights,
U6		Apps for somatic activities and regulation	Yoga, walking, work, journaling, reflecting, spend time with family, travelling
U7		How We Feel, Apple activity tracking	Physical activities, socializing, being outdoors, walking my dogs, camping, cooking, dancing, reading

Participant	Years of experience	Technology usage with clients	Meaningful activities
OT1	5	Quite often	Walking, reading, cooking, shower after workout, going to the spa or sauna
OT2	1	Most of the time	Going to the gym, go outside, spend time with niece and nephew, going to the park
OT3	2	Sometimes	Working with kids, music, singing, social connections
OT4	20	Most of the time	Playing with my son, hiking
OT5	10	Never	Kayaking, rock climbing, walking my dog, play piano, painting project
OT6	32	Most of the time	Work, time with friends and family, travelling
OT7	22	N/A	Walking in the forest, playing volleyball and tennis, meditating, sauna, teaching, solving riddles, dancing, massaging

U, Individuals with lived experience of mental health challenges; OT, occupational therapist

### 3.2 Which aspects of activity participation should be visualized in digital twins

**Figure 2:**
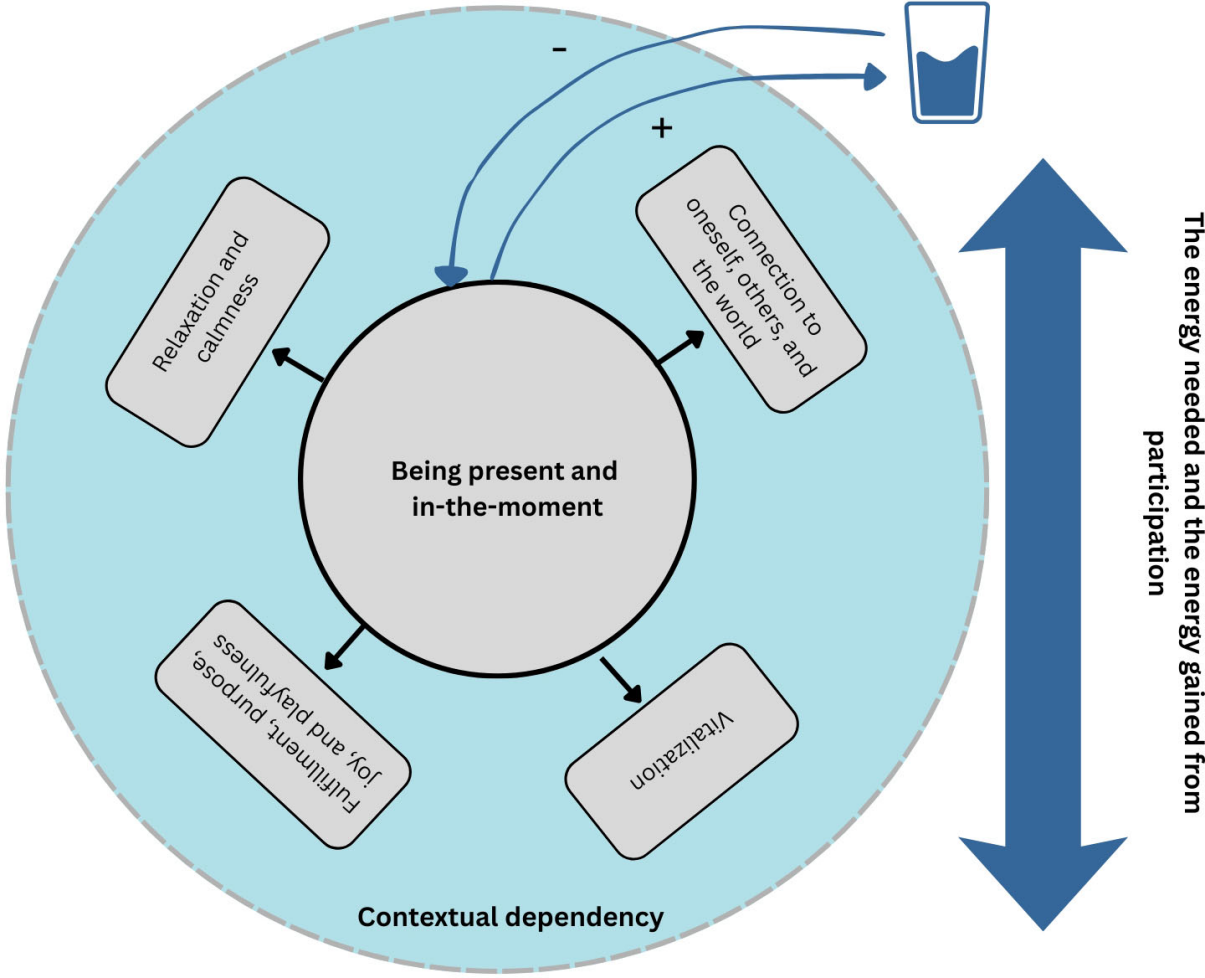
Categories representing ‘participation in meaningful activities’ in digital twins.

The analysis revealed 4 categories representing aspects of participants’ participation in meaningful activities, which they wished to have visualized in participation-focused digital twins. Categories were related to each other and were seen to influence each other as illustrated in **Figure 2**. The first category included the level of ‘being present and in-the-moment’, which participants described as the core aspect of their participation in meaningful activities. The second category focused on ‘related or resulting experiences of being present and in-the-moment’ consisting of 4 subcategories, including 1) the level of experiencing relaxation and calmness, 2) the level of experiencing fulfillment, purpose, joy, and playfulness, 3) the level of feeling vitalized, and 4) the level of experiencing connection with oneself, others and the world. The third category focused on ‘the energy needed and the energy gained from participation’ which participants perceived as an important aspect to be visualized in participation-focused digital twins. This included visualizing the concept of “starting energy” as well as the varying levels of energy required by and gained from participation in meaningful activities. The fourth category centered around ‘the influence of the context”, which participants highlighted as an important part for visualizing and understanding their participating in meaningful activities in digital twins.

#### 3.2.1 Level of experiencing being present and in-the-moment

Participants described how being fully present and in-the-moment with the activity was an important indicator for them to know how much they were involved in a meaningful activity. OT3 described it as “putting yourself into the whole activity”, having the “mind [ … ] engaged in it”, and “just focus on what’s right in front of me and kind of the environment that I am in”. Some also described it as their level of “sense of flow” (U1) or “calm flow” (U6), and U4 described this experience as “just forgetting myself in the mood, moment of doing stuff”. Being present and in-the-moment was also described as disconnecting from worries, thoughts, anxiety, things that need to be done, and “thoughts of responsibility” (U4). U7 described it as “during the moments, sometimes I notice, that I’m not, I’m not thinking of the stressors that [I] normally [do]. [ … ] they’re kind of on the back burner.”

However, one participant highlighted that ‘just’ being present in the moment alone does not fully capture their experienced participation in meaningful activities and may even be misleading: “But I am also very engaged [present in the moment] when somebody is questioning me or having an argument with me. So, after an argument, I’ll be very drained out for sure” (U3). This participant illustrated how being present and in-the-moment needed to be accompanied with additional aspects, related and resulting experiences, to fully capture and visualize individuals’ participation in meaningful activities (i.e., those that carry individual significance and simultaneously generate positive affect and energy) in a digital twin.

#### 3.2.2 Related or resulting experiences of being present, in-the-moment

This category consisted of 4 subcategories that described related and resulting experiences of being involved in meaningful activities that complement the first category of being present and in-the moment and can last for longer than that in-the-moment experience. These subcategories included: 1) the level of experiencing relaxation and calmness, 2) the level of experiencing fulfillment, purpose, joy, and playfulness, 3) the level of feeling vitalized, and 4) the level of experiencing connection with oneself, others and the world.

##### 3.2.2.1 Level of experiencing relaxation and calmness

Experiencing relaxation and calmness was described by participants as “restorative” (U1), and “a little bit peaceful” (OT1). Some participants experienced relaxation and calmness “during the activity” (OT7), whereas others described it as an experience also after participating in a meaningful activity, such as illustrated by U4 “it makes me relaxed immediately, and then I continue to be a bit more relaxed”.

For a digital twin to visualize aspects of participation in meaningful activities, some participants remarked the importance of separating the experience of relaxation and calmness that replenishes energy from those experiences that may be perceived as rest but do not replenish energy, as U3 illustrates: “I am a little bit addicted to Netflix and kind of like just sitting a little bit and watching stuff. In my mind, that was giving me energy, but I learned [ … ] that it’s a passive rest, that it’s not replenishing my energy.”

##### 3.2.2.2 Level of experiencing fulfillment, purpose, joy, and playfulness

Experiencing fulfillment, purpose, joy, and playfulness was described as “things that fulfill you and give, you know, give meaning and reason and bring happiness and give you energy” (OT1), an experience of “feeding my soul” (OT5), “the effort was not useless” (OT6), “feeling lightness and like joy and maybe just like playfulness” (OT7), and being “more grateful” (U3). Fun as well as happiness were also mentioned, however, not all participants liked those concepts for the visualization of their participation in meaningful activities in a digital twin, as illustrated by U4 “I am definitely happy afterwards, but I wouldn’t describe it as fun” or U3 “I don’t relate so much with the word happiness, because I don’t understand what it is”.

##### 3.2.2.3 Level of feeling vitalized

Experiencing a feeling of vitalization was described as “a sort of buzz, like I can feel it in my body” (U1), “mental energy” (OT1), and “be like, yeah, you know, hyped” (U1). This experience was described, for example, in relation to physical activities “that [are] obviously physically tiring, but I do find it helps my energy overall” (U1).

The importance of visualizing the feeling of vitalization or mental energy in a participation-focused digital twin was illustrated by U4: “If I feel happy but don’t have mental energy, I am soon going to be sad because anxiety is going to build up because I am not going to get stuff done. If I feel sad but have mental energy, I am soon going to feel happy because I am going to be able to take care of the things. So, I think energy or energeticness is probably the most important part.”

##### 3.2.2.4 Level of experiencing connection with oneself, others, and the world

Experiencing connection to oneself and others was described by participants as “sort of being in my body more” (U1), “can sort my own thoughts” (U4), “[a] deep sense of connection with God” (U2), and “bonding together through reading. Yeah, I think that’s how it brings that joy and that energy”. The connection with others was also described through music as illustrated by U7, “you are connecting with like a song, and you feel a sense of like oh somebody knows, like what I am going through or feeling, and that feels like oh okay weight off your shoulders”.

The connection to the world was often described in relation to activities in the nature as illustrated by U7 “Yet it can give you such a feeling of connectedness and maybe gratitude even for, like just the beauty or I like to walk by a pond where there’s always lots of birds and ducks and it just helps me to slow down and to appreciate.” Similarly, OT7 described this connection as being “humble about myself, because the nature is there [ … ] there are big trees [ … ], the energy of 40, 50 years [ … ]. I am just a tiny piece in the whole world. And this is a very humbling thing.”

#### 3.2.3 The energy needed and the energy gained from participation

Participants mentioned the importance of having energy to participate in an activity to be able to receive energy back from the participation experience in meaningful activities and wanted to see that level in a participation-focused digital twin. Depending on that starting energy level, participation in different activities was perceived as more likely to succeed and give energy and recover from stress as illustrated by U2: “for me, its almost like, as if I have two avenues, like if I’m really low in energy, it would be things that don’t involve a lot of people and don’t involve a lot of probably physical exercise. For example, I would go for a walk, listening to some music. [ … ] And if I have more energy, probably I would go to the other routes, which is like going to the gym.” Two types of activities were also described by U4: “So let’s split it into two things that I find inherently fun and easy to do when it gets started, like playing board games with friends or playing a computer game. And then the other group of activities are exercise and doing things that I like, but which take effort, like writing a story or performing an otherwise exciting work task [ … ] I don’t have the emotional excitement, but my brain knows that I will like it when I either get done with it or when I do it.”

#### 3.2.4 The influence of the context

Participants mentioned the importance to consider contextual and environmental factors in a digital twin when capturing and visualizing individuals’ experience of participation in activities. For example, U2 stated: “I think the same activities that sometimes give me energy can also use up my energy depending on how I am”. Similarly, OT4 reflected: “it [whether it results in positive energy or not] really depends on other factors. Like, for example, who am I hiking with? What load? How far? Where? When?” and OT3 “the vibe is very important to me [ … ]. It was not only like what we were doing, we were literally just sitting there.”

They reflected on how the experience of participation was an in-the-moment experience that could change immediately depending on the context. At the same time, they described participation as something important to look over time and across activities, illustrated by U5 “And then you can get on a positive roll where you do some chores, then maybe you go for a walk and then you play games, and everything is more fun”. U2 described it as “it’s almost like an ecosystem where we are in the middle and then there are other circles that kind of like surround us. So you have the people who are really close to you, the environment and things that are really connected to you [ … ]. It feels like streams of things that would change how this activity will give me energy.”

### 3.3 How should these aspects of activity participation be presented in digital twins

The analysis revealed 2 categories related to how identified aspects to represent activity participation should be visualized in digital twins: 1) Symbols and charts to visualize the now-situation, and 2) graphs for visualization over time.

#### 3.3.1 Symbols and charts to visualize the now-situation

Participants described various visual elements such as point systems, pie charts, and symbolic representations to visualize aspects of their activity participation in digital twins. Suggested symbols included thermometers, bubbles of water, candles, pendulums, batteries, and heartbeats. Only two participants mentioned using a visual representation of themselves to reflect their experiences of activity participation in digital twins.

Visual elements were often framed as indicators of the level of participation experiences, particularly with regard to energy dynamics. For example, some participants described the visualization as reflecting a ‘energy household’, representing both expenditure and replenishment. As OT3 explained, this could take the form of a “dual arrow [ … ]. I am giving [ … ] something and theoretically I’m getting something back from it, whether it’s the activity itself, whether it’s the people in my environment, you know, but its kind of the back and forth”. Similarly, U2 referred to an “image of the battery”, stating, “I was like almost 0% last week and then I charged this weekend until 15. And then because I delivered this lecture, I went back to 5%.”

Color was also frequently highlighted as a key component of effective visualization, with the emphasis that it should “stay the same” (OT3) throughout the tool. The most commonly mentioned color scheme was based on traffic light colors: green signified high energy levels, and red signified low energy. These colors were also suggested to be applied to categorize activities: Green representing activities that give energy and that “you can do all day” (OT1) and red signifying draining activities that “cannot [be] continue[d] for very long” (OT1).

#### 3.3.2 Graphs for visualization over time

All participants expressed the desire to visualize their experiences of participation in meaningful activities over time, primarily through the use of graphs. As OT1 stated, “I would like to see how I’m doing, my energy across the week”. Such visualizations were perceived as useful for identifying patterns between activities and associated experiences. U1 elaborated: “the visualization [ … ] it helps you sort of zoom out and see the big picture and do some kind of perspective taking as well as seeing those patterns and things.”

Participants noted the challenge of accurately remembering and assessing experiences and feelings without documentation. U1 reflected, “I think it’s really easy to think, [ … ] I’ve had a rubbish day [ … ] and I will forget that that’s just an instance in time. [ … ] Tomorrow might be great.”

Most participants envisioned simple, two-dimensional graphs, where the x-axis represented time and the y-axis represented a specific experience or feeling related to their participation in meaningful activities. OT5 expanded on this idea by proposing charts that reflect time spent within an ‘optimal participation zone’ For example, a graph could indicate “this week you hit your optimal zone or stayed within that zone on average 50% of the time, 30% of the time.”

## 4 Discussion

Participation in meaningful activities is an important indicator of health and well-being (17) and a key outcome as well as intervention target in mental health occupational therapy (1). Digital twins may offer a promising way to support individuals with mental health challenges in their participation in meaningful activities. However, little is currently known about how digital twins could represent individuals’ participation in meaningful activities. The aim of this study was therefore to explore the perspectives of occupational therapists and of individuals with lived experience of mental health challenges regarding which aspects of participation in meaningful activities (i.e., those that carry individual significance and simultaneously generate positive affect and energy) should be visualized and how they could be represented in digital twins.

### 4.1 Which aspects of activity participation should be visualized in digital twins

Our results identified four main aspects that participants considered important to visualize in digital twins focusing on participation in meaningful activities. Those aspects included individuals’ level of ‘being present and in-the-moment’, ‘related or resulting experiences of being present and in-the-moment’ (relaxation and calmness; fulfillment, purpose, joy, and playfulness; feeling vitalized; connection with oneself, others and the world), ‘the energy needed and the energy gained from participation’, and ‘the influence of the context’.

The reported experience of ‘being present and in-the-moment’ aligns closely with the flow theory (25, 26), literature on experiences related to recovering from stress and exhaustion (27), and literature on mindfulness (28). Flow theory describes flow as “the state in which people are so involved in an activity that nothing else seems to matter; the experience itself is so enjoyable that people will do it even at great cost, for the sheer sake of doing it” (25, p. 4). Similarly, in recovery literature (27, 29), ‘being present and in-the-moment’ aligns with the concept of psychological detachment, which refers to the ability to mentally distance oneself from daily stressors, for example, by focusing on a task that fosters immersion and temporarily blocks out external concerns. Whereas flow theory and literature on recovery experiences emphasize positive, pleasurable states, mindfulness can arise in both, positive and negative contexts. It has been defined as ‘the awareness that emerges through paying attention on purpose, in the present moment, and nonjudgmentally to the unfolding of experience moment by moment’ (28).

Similar to the description of mindfulness, our findings also suggest that the experience of ‘being present and in-the-moment’ can occur with equal intensity during both pleasant and unpleasant activities. This echoes research on the experience of involvement in daily life activities among children and youth with childhood-onset disabilities (30, 31). High levels of ‘being present and in-the-moment’ during unpleasant activities, such as an argument, often result in a loss of energy rather than a gain. This highlights a potential limitation in using ‘being present and in-the-moment’ as a stand-alone indicator for representing and visualizing experiences related to participation in meaningful activities (i.e., those that carry individual significance and simultaneously generate positive affect and energy) in digital twins. To address this, participants emphasized the importance of representing and visualizing additional, related experiences including relaxation and calmness; fulfillment, purpose, joy, and playfulness; feeling vitalized; and a sense of connection with oneself, others and the world. These experiences align with, and may offer additional insight into, the occupational elements of ‘occupational experience’ and ‘participation’ described in the Transactional Model of Occupation (TMO), a conceptual model within occupational therapy (1). Occupational experience refers to a person’s subjective experience of doing (including attending) and encompasses as wide range of positive to negative responses. Participation is described as emerging “when doing is combined with an experience of value in that doing” (1). The TMO portrays participation as involving experiences such as contributing, making a difference, and being helpful – experiences that resonate with the themes of purpose, fulfillment, and potentially joy or playfulness identified in our study. It also includes feeling included, accepted, and connected, which parallels our findings related to feeling connected to oneself, other and the world. Experiences such as feeling vitalized, relaxed and calm showed less overlap, perhaps due to the nature of the activities examined in this study, which primarily focused on those that generate energy. However, how these experiences fully map onto the occupational elements within the (1) TMO warrants future research.

The related experiences ‘relaxation and calmness’; ‘fulfillment, purpose, joy, and playfulness’; ‘feeling vitalized’; and ‘a sense of connection with oneself, others and the world’ also correspond to the following recovery experiences described in the literature (27): ‘Relaxation and calmness’ aligns with the recovery experience ‘relaxation’, while experiences of fulfillment, purpose, joy, and playfulness and vitality may partially overlap with ‘mastery’. Mastery refers to a personal sense of success in engaging with an activity, often associated with opportunities for learning and growth (27). While fulfillment, purpose, joy, playfulness, and vitality can indeed result from learning and growth, activities may also evoke these experiences in other ways, independent of such processes. The one recovery experience that was not aligned with our results was ‘control’. According to Sonnentag et al. (27), ‘control’ refers to the degree of autonomy in choosing what to do. While choice is considered an important influencing factor of participation in activities, it is not typically viewed as a direct experience of participation (8), which may explain its absence from our results.

Our results suggest representing both the energy required for and gained from participation, which aligns with prior research reporting on recovery activities and experiences of people with a history of exhaustion disorder (4). Similar to our results, this study highlights the need for a certain amount of energy to do certain types of recovery activities. For example, physical activity with higher intensity was only experienced as recovering when there was enough ‘starting energy’ (4). In a participation-focused digital twin, tracking these energy exchanges could help individuals recognize when they are nearing a threshold where initiating participation in energy-restoring activities becomes difficult, potentially offering preventive benefits to be explored in future studies.

Aligned with a growing body of evidence (32–34), participants repeatedly emphasized the influence of contextual factors on the participation experiences, as well as the complexity of identifying patterns between activities and participation experiences when considering contextual variability. Even seemingly minor contextual changes can substantially alter the situation and its associated participation experiences, which sometimes happens in an instance. For example, the arrival of a new person during an activity may completely change the nature of the participation experience. While there is strong evidence supporting the significant role of context in shaping participation (32–34), less is known about the specific dynamics of contextual factors and participation experiences. In occupational therapy literature, context has been defined as comprising temporal, sociocultural, geopolitical, and physical and social environmental elements (1). Future research could explore how such interactions might be modeled in a participation-focused digital twin and how a digital coach could help individuals reflect on these patterns to better understand their experiences and the role of the activities and the context.

### 4.2 How should these aspects of activity participation be presented in digital twins

Our results revealed two categories for how aspects of participation in meaningful activities should be visualized: ‘symbols and charts to visualize the now-situation’, and ‘graphs for visualization over time’. These categories reflect participants’ emphasis on both immediate feedback and longitudinal monitoring. For the “now” situation, participants suggested point systems, pie charts, and symbolic representations, such as thermometers, water bubbles, candles, pendulums, batteries, and heartbeats to indicate levels of participation and energy dynamics. Interestingly, only two participants mentioned using a visual representation of themselves to reflect their experiences of activity participation in digital twins. In human–computer interaction and health informatics, avatar-based or photorealistic self-representations are often assumed to foster identification, motivation, and empathy with one’s own data (35, 36). However, the present findings suggest that, in the context of activity participation and energy tracking, users may find abstract metaphors (e.g., batteries, thermometers) more fitting. This divergence could be explained by the fact that participation cannot be fully observed from the outside (8), unlike other body structures (e.g., organs) that have concrete, widely recognized visual forms.

For visualizing participation over time, participants favored simple, two-dimensional graphs mapping time against a selected measure of participation experience or energy. This approach was valued for its capacity to reveal patterns, support perspective-taking, and counteract the distortions of short-term recall. Some participants proposed tracking time spent in an “optimal participation zone”. The idea aligns with findings from a scoping review exploring the concept of ‘involvement in daily life activities’ from the perspective of children and youth with childhood-onset disabilities (30). In that review, experience of involvement was described across five levels (i.e., non-involvement, partial involvement, full involvement, hooked involvement, involvement overload), of which levels 2 and 3 might correspond to an ‘optimal involvement zone’. Levels 1, 4, and 5 were described as either non-involvement or too much involvement, often resulting in reduced enjoyment. Integrating this with broader self-regulation literature, an “optimal participation zone” resonates with an optimal zone of regulation (green zone) within the “Zones of Regulations” framework, a tool to recognize and communicate feelings and state of emotions (37). This zone is descried as “the optimum state [ … ] and includes feelings such as: happy, calm, focused, proud” (37). Outside this zone, under- or over-engagement may compromise both regulation and the quality of participation. However, what constitutes an optimal participation zone and how it fluctuates across individuals and contexts remain open questions for future research.

### 4.3 Limitations

Results from this study should be interpreted in light of several limitations. First, all participants (individuals who identify as someone with mental health challenges and occupational therapists) were highly educated, with at least a master’s degree. This homogeneity in educational background may have introduced bias of the findings. For example, highly educated individuals may be more inclined to visualize data as graphs or charts. Thus, further research with a more diverse sample is needed. Second, we did not collect data regarding participants’ specific diagnosis, the severity of their mental health challenges, or what part of recovery they were in, which may have resulted in a heterogenous sample with potentially diverse needs and perspectives. Third, we did not collect information about the work setting of the interviewed occupational therapists, nor did we require practical experience in mental health occupational therapy for participation. These factors may have limited our ability to contextualize or compare their perspectives. Nevertheless, we achieved data saturation, which strengthens the credibility of the findings despite these limitations.

## 5 Conclusion

This study explored the perspectives of occupational therapists and individuals with lived experience of mental health challenges regarding which aspects of participation in meaningful activities should be visualized and how they could be represented in digital twins. Key aspects identified for visualization include being present and in-the-moment, related or resulting experiences of being present and in-the-moment, the energy needed and the energy gained from participation, and the influence of the context. Our findings suggest that abstract metaphors and simple graphical representations may be more suitable than direct self-images for capturing these dynamic experiences. Future research should further investigate how contextual factors can be dynamically modeled, how a digital coach might support with reflections about participation patterns, and how optimal participation zones vary across individuals to refine digital twin applications in this field.

## Data Availability

The raw data supporting the conclusions of this article will be made available by the authors, without undue reservation.

## Footnotes

1 https://pub.dev/packages/fl_chart
